# Thoughts on Surgery, Operative and Conservative, Suggested by a Visit to the Battle-Field and Hospitals of the Army of Tennessee

**Published:** 1864-09

**Authors:** G. M. B. Maughs

**Affiliations:** P. A. C. S.


### CONFEDERATE STATES
### MEDICAL & SURGICAL
### JOURNAL.

Vol. I. RICHMOND, SEPTEMBER, 1864. No. 9.

### ORIGINAL COMMUNICATIONS.

√ Art. I.—*Thoughts on Surgery, Operative and Conservative, suggested by a visit to the Battle-field and Hospitals of the Army of Tennessee.* By G. M. B. Maughs, P. A. C. S.

That a great desire should exist upon the part of hundreds of young physicians, who by the exigencies of the times had suddenly been transformed into army surgeons, to perform operations was most natural. And that many limbs should have been sacrificed to the want of, and desire to obtain, experience during the early periods of the war, was to be expected. But, with all these chances against the retention of a wounded limb, we might reasonably suppose that three years continuance of the most destructive war of modern times, with the thousands of mutilated men all over the land—with thousands more who have lost their lives by, or in despite of, the operations to which they have been subjected—would have sufficed to gratify the penchant for lopping off limbs with even the most ambitious. That it has not done so, however, will be apparent to any surgeon who will visit the battle-field, where cases of compound fracture are condemned to the knife with as little hesitancy as if men's limbs, like those of the salamander, were reproduced with great certainty.

Indeed, we fear that the highest boast of modern surgery, its conservation, whereby limbs that formerly would have been condemned are now preserved, has been lost sight of, in the desire, unfortunately too common, to make the most out of the present surroundings—the preservation of life and limb being made subordinate to the great end and aim of the army surgeon, the acquiring experience in capital operations. And this, within due bounds, laudable desire to become skilled in their profession is, unfortunately, by no means confined to the field-surgeon; for, should the sufferer leave the field with a limb, through which a ball had passed, he cannot long congratulate himself upon his good fortune in not having been subjected to the frightful hazards of an amputation; for on arriving at the hospital he has another ordeal, fiercer perhaps than that through which he has just passed, as here he meets with one or half a dozen surgeons and assistant surgeons, zealous in the cause of humanity, all anxious to relieve him of so useless an appendage. These hospital surgeons, with their assistants, are even less likely to let pass such an opportunity of acquiring or displaying skill than field-surgeons, from the fact that they less frequently have so rich a morceau thrown them; and the knowledge of the fact that amputation will almost certainly be performed at the hospital, acts as a constant, and to some extent justifiable, inducement to the field surgeon to amputate all limbs *that will bear cutting off.* And when, from either the small extent of the wound or the part in which it occurs, the idea of amputation cannot be entertained, *resection* or *excision* frequently comes to the surgeon's relief, as almost all cases of compound fracture will admit more or less interference in this way. And good cases for acquiring or displaying skill in this modern development, of what was intended to be conservative surgery, too frequently present themselves, where, with some considerable patience and industry, by dissecting up from one to three inches above any injury to bone or soft parts, the articulating ligament may be pierced, and the *entirely sound* head of the bone turned out. It is true, by this operation the dangers to life have been greatly increased, and the probabilities, when the patient survives the operation, of the limb ever again being useful, immeasurably diminished; but then it gives *resection* a chance if the *limb* has none. But it will happen occasionally, either from the nature of the wound or its great distance from an articulating cavity, that resection cannot be resorted to. What then is to be done? Remove the loose spiculæ and leave the cure to nature? By no means; for though it is not doubted a cure would be effected, yet it might be thought to show the paucity of the surgeon's resources, and *excision* fortunately comes to his aid in this, as resection had in the other condition. And that surgeon must be a novice indeed who—with the aid of free incisions, by carefully dissecting from the periosteum and soft parts all partially detached pieces of bone—cannot produce a lesion of two or three inches in the continuity of the shaft of a bone. What if nature, overtaxed and placed at disadvantage, is unable, as is too frequently the case, to restore the continuity—to fill up this hiatus with ossific deposit? Well, the surgeon has, at least, demonstrated that, however bad the wound may be, art can make it worse. It would seem scarcely necessary to state that such practice is an unwarrantable interference with, and tax upon, the reproductive energies of the individual—utterly abhorrent to scientific, conservative treatment, and receives no support from the modern views of the physiological development of bone.

In the introduction of resection and excision into modern surgery, it was intended they should be conservators, superseding, in many cases, amputations. The former may be resorted to in cases where the head of the bone is shattered; but in all cases, where the fracture is below the anatomical neck, and where the articulating ligament, anatomical neck and head of the bone are intact, resection is meddlesome surgery, increasing the risks to life and diminishing the chances for a useful limb, while excision of any considerable portion of the shaft of a bone is a dangerous expedient, scarcely warranted by the results of the practice, and should be resorted to with great caution and in extremely rare cases.

In cases of compound fracture of the shaft of a bone, where amputation has been decided against, it would be well to remove all entirely detached pieces of bone lying in, and immediately contiguous to, the wound, as should be done with other foreign bodies; but all pieces not immediately in or at the tract of the ball, and that are adherent to their periosteum and the soft parts, should be let alone, as they serve as points for the development of ossific matter, thereby securing a bony connection, and greatly lessening the time required for the reparation of the injury.

As to amputations, the most serious and most abused of field operations, it may be well to inquire whether, *except in cases where the limb has been carried away, leaving an irregular stump, or where there is very great destruction of the soft parts, with injury to the large blood-vessels and nerves,* it would not be well for the cause of humanity were these operations upon soldiers in the field entirely proscribed, and the surgeon's case, except that of the chief surgeons of divisions, left as bare of amputating knives as a Federal medicine chest is of calomel and tartar emetic, and for the same reason.

On examining this subject, we shall consider the frightful dangers to life of amputations of the thigh, and, by comparison with the results of wounds of the same class treated without amputations, establish, as we believe, the superiority of conservative over operative surgery; and if it can be shown that, by an attempt to save the limb, the dangers to life are lessened, the claims of such practice need but little further argument, as most persons, we opine, are ready to admit that a natural limb, though a little shorter, is greatly more useful than any artificial one could be; and if any one has a desire to become expert in operative surgery, a glance at the statistics of amputations of the thigh, with their frightful percentage of mortality, may well cause him to hesitate to acquire experience at such a fearful loss of life.

By reference to any or all statistics, both of foreign wars and the present one, it will be found that, independent it may be of the nature of the wounds, more than half of all amputations at or above the knee prove fatal, and in the upper-third of the thigh this alarming mortality runs up to eight or nine out of ten; that is, out of every hundred so operated upon, eighty or ninety will die; and this result would not, most probably, be greatly changed were such amputations performed upon limbs previously uninjured, so abhorrent is nature in the higher animals to mutilations. As it is thus apparent that we must kill our patients by cutting off their limbs, it becomes a question of morals, as well as science; and if a mode of treatment less destructive of life can be adopted, should become one of law, whether we be allowed thus recklessly to sacrifice the lives of our brave soldiers. At all events, when it is considered that from sixty to ninety percentage of all soldiers thus mutilated will die, it becomes a question of too much gravity to be left to the whim, passion or judgment of an inexperienced surgeon or assistant; but that, at least, this much of a safeguard be thrown around the wounded soldier, that every army division have a committee of at least two of the most learned and experienced of its surgeons, chosen by the surgeons and assistant surgeons of the division, whose duty it shall be to consult with the surgeon or assistant in charge, and without whose advice and consent, except in extreme cases, it should not be lawful to amputate a limb.

To all surgeons of experience in the army, the conservative treatment of these cases will find support in the recollection of numerous cases, where, from some cause, as the opposition of the patient, the presence of persistent shock, or the conservative views of the surgeon, the limb has been spared and recovery has taken place in many instances, without a bad symptom and in a surprisingly short time. I have witnessed a number of such cases, and have never conversed with an army surgeon of experience (though amputation is the rule) who cannot refer to a greater or less number. Among less notable cases, Governor Clark and Col. Holden (now member of Congress) may be cited, and, as both these gentlemen's limbs were fractured in the upper-third, had amputation been performed, there is little doubt that a newspaper paragraph, announcing their devotion and heroism, would have been substituted for their present valuable services. And this limited support, which the conservative treatment receives from the observations of myself and others, is not *only in accordance with all standard authorities on surgery for the last quarter of a century,* but is fully supported by the result of cases occurring in the Richmond hospitals during a given period, where, of 172 amputations of the thigh, with almost all of them at points most favorable, (10 of the upper, 28 of the middle and 70 of the lower-third, with 64 the seat not given, which gives, by calculation, 16 of the upper, 44 of the middle and 112 of the lower-third,) we have 103 or 60 per cent. of deaths. Now, it will be noticed that, notwithstanding the bill of 60 per cent. mortality, nearly all of these amputations were at points most favorable—the lower and middle-thirds—consequently must have been for injuries at or near the knee. What it would have been had the operations been performed for wounds received at these points will be made apparent in considering the conservative treatment.

In these hospitals, during the same time, there were treated for similar wounds, compound fracture—gun-shot wounds—of the thigh, without amputation, 216 cases. Of these, 83 were in the upper, 57 in the middle and 38 in the lower-third—the seat of 38 is not given. By calculation, we have 100 of these 216 cases in the upper, 69 in the middle and 47 in the lower-third. Of this 100, as the ball had passed through the upper portion of the bone, at or through the trochanters, in many instances, at least one-third would have required disarticulation at the hip-joint—an operation always fatal. The other two-thirds would have required amputation near or through the trochanters—an operation but little better than one between the first and seventh cervical vertebræ, as 95 per cent. of such cases prove fatal. Of the first 100, therefore, had amputation been performed, not more than three or four would have recovered. Of the remaining 116 cases, most of which were injuries of the middle-third, and, consequently, in many cases, would have required amputation in the upper-third, we may safely calculate the average mortality at 76 per cent. or 28 recoveries—giving 32 recoveries to 184 deaths. Now what was the result of conservative treatment? Out of the 216 cases, 80 recovered and 121 died—leaving 15 unaccounted for. By calculation, we have 86 recoveries and 30 deaths, or nearly 40 per cent. recoveries in conservative, against less than 15 per cent. in operative surgery. A result in favor of life surely worth regarding, to say nothing of the advantages, in most cases, of a natural over an artificial limb. The last point may, however, in this mechanical age, be a mooted one, as there are not wanting advocates of artificial limbs who would insist upon their superiority over natural ones. We are not, however, of the number, except in cases where the surgeon was anxious to acquire experience.

I am aware that it has been stated, I know not upon what authority, that this immense superiority of conservative over operative surgery, taken from the Richmond hospital records, is not supported by results attained elsewhere, and, to account for it, it has been suggested that many cases may have died unreported. This may be a fortunate guess; but might it not be equally so to guess the same thing has occurred, to even a greater extent, in cases of amputation? Nor can it be said that the amputated cases were the least serious ones; their locality proves the reverse, and, indeed, reveals the secret why so many were allowed a chance for life and limb.

